# Dr. William Hailes

**Published:** 1912-08

**Authors:** 


### OBITUARY

---

Dr. William Hailes, Albany Medical College, 1870, died July 6, aged 62. He was Prof. Emeritus of Pathologic Anatomy and Histology in his alma mater and Attending Surgeon to the Albany and St. Peter's Hospitals.

